# Effects of Dietary Lipid Composition and Fatty Acid Desaturase 2 Expression in Broodstock Gilthead Sea Bream on Lipid Metabolism-Related Genes and Methylation of the *fads2* Gene Promoter in Their Offspring

**DOI:** 10.3390/ijms20246250

**Published:** 2019-12-11

**Authors:** Serhat Turkmen, Erick Perera, Maria J. Zamorano, Paula Simó-Mirabet, Hanlin Xu, Jaume Pérez-Sánchez, Marisol Izquierdo

**Affiliations:** 1 Aquaculture Research Group (GIA), IU-ECOAQUA, Universidad de Las Palmas de Gran Canaria, Crta. Taliarte s/n, 35214 Telde, Spain; mariajesus.zamorano@ulpgc.es (M.J.Z.); hanlinxuulpgc@outlook.com (H.X.); marisol.izquierdo@ulpgc.es (M.I.); 2 Department of Biology, University of Alabama at Birmingham, 35294, Birmingham, AL 35294, USA; 3 Nutrigenomics and Fish Growth Endocrinology Group, Institute of Aquaculture Torre de la Sal, IATS-CSIC, Ribera de Cabanes s/n, 12595 Castellón, Spain; erick.perera@csic.es (E.P.); paula.simo@csic.es (P.S.-M.); jaime.perez.sanchez@csic.es (J.P.-S.)

**Keywords:** DNA methylation, digital droplet PCR, modulation of lipid metabolism, nutritional programming, parental nutrition

## Abstract

Polyunsaturated fatty acids (PUFA) in parental diets play a key role in regulating *n-3* LC-PUFA metabolism of the offspring. However, it is not clear whether this metabolic regulation is driven by the precursors presented in the diet or by the parental ability to synthesize them. To elucidate this, broodstocks of gilthead sea bream with different blood expression levels of *fads2*, which encodes for the rate-limiting enzyme in the *n-3* LC-PUFA synthesis pathway, were fed either a diet supplemented with alpha-linolenic acid (ALA, 18:3*n-3*) or a control diet. The progenies obtained from these four experimental groups were then challenged with a low LC-PUFA diet at the juvenile stage. Results showed that the offspring from parents with high *fads2* expression presented higher growth and improved utilization of low *n-3* LC-PUFA diets compared to the offspring from parents with low *fads2* expression. Besides, an ALA-rich diet during the gametogenesis caused negative effects on the growth of the offspring. The epigenetic analysis demonstrated that methylation in the promoter of *fads2* of the offspring was correlated with the parental *fads2* expression levels and type of the broodstock diet.

## 1. Introduction

Aquaculture is the fastest growing food production sector with great potential to supply high-quality food for the expanding world population [1,2]. One of the major challenges for the aquaculture industry is to secure feed ingredients of marine origin, fishmeal (FM), and fish oil (FO) that supply high quality protein and lipid [3,4,5,6]. In fact, capture fisheries—including that for production of FM and FO—remain steady over the last decade [1]. Both FM and FO are ingredients with a high nutrient bioavailability and adequate nutritional composition, which fulfill essential amino acids and fatty acids requirements of fish species [7,8]. Alternatively, oils and meals obtained from terrestrial crops, such as plant based protein sourses (VM) and vegetable oils (VO), are currently used to replace FM and FO in fish feeds [7,8]. Complete substitution of FO by VO adversely affects fish immune system and stress and disease resistance [3,4,5,6]. In addition, it also reduces the muscle content of long-chain *n-3* polyunsaturated fatty acids (*n-3* LC-PUFA), such as EPA (20:5*n-3*) and DHA (22:6*n-3*) that negatively affect the nutritional value of farmed fish for humans [4,9,10,11,12].

Most fish cannot synthesize *n-3* and *n-6* PUFA *de novo* and they must be supplied in the diet. Generally, essential fatty acid (EFA) requirements of freshwater fish can be met by the supply of 18:3*n-3* and 18:2*n-6* fatty acids in their diets, whereas the EFA requirement of marine fish can only be met by supplying the LC-PUFA, EPA, and DHA [13]. Unlike freshwater fish, marine fish either lack or show a low activity of Δ6-desaturase, and thus require the long chain PUFA’s, EPA, and DHA to meet their EFA requirement. The bioconversion of 18:3*n-3* to EPA and DHA involves desaturations at Δ-6 and Δ-5 positions in the carbon backbone and an intermediate 2-carbon chain elongation step. Synthesis of DHA from EPA requires elongation of EPA to 22:5*n-3* and 24:5*n-3* which is then converted to 24:5*n-3* and 24:6*n-3* by Δ6-desaturase and finally shortened to DHA by β-oxidation. Among enzymes involved in *n-3* LC-PUFA synthesis, Δ-6-desaturase enzyme (Fads2), encoded by *fads2* gene, is considered to be the rate-limiting step in the biosynthetic pathway of PUFA in gilthead sea bream [14,15]. LC-PUFA synthesis also involves chain elongation catalyzed by elongases (Elovl) with different substrate preferences [16]. Among them Elovl6 is a key lipogenic enzyme that elongates long-chain saturated and monounsaturated fatty acids of 12, 14, and 16C, and has received much attention due to its link with certain metabolic disorders [17]. LC-PUFA may have a direct effect on the expression of other genes related to lipid or carbohydrate metabolism [18]. For instance, lipoprotein lipase (Lpl) facilitates the tissue uptake of circulating fatty acids [19] from lipoproteins and *lpl* gene expression in the liver can be regulated by *n-3* PUFAs [20]. Also, the energy supplied by β-oxidation of free fatty acids is transported into the mitochondria in the form of fatty acyl-carnitine esters by carnitine acyltransferases such as carnitine palmitoyltransferases (Cpt) [21]. Replacement of FO by VO changes the fatty acid composition of liver and muscle, affects the β-oxidation capacity as well as regulates the expression of *cptI* and *cptII* genes [22,23,24,25]. β-oxidation also takes place in the peroxisome, which is modulated by peroxisome proliferator activator receptors (Ppars). Three different ppar isoforms (𝛼,β,𝜸) have been characterised in gilthead sea bream, *ppar𝛼* being the major form expressed in the liver [26]. Ppars are nuclear receptors that regulate differentiation, growth, and metabolism, and epigenetic mechanisms have been described to regulate these processes in mammals [27]. For example, feeding pregnant rats a protein-restricted diet reduces methylation of the Ppar𝛼 promoter in the offspring, and hypomethylation persists into adulthood [28]. Another gene considered to be potentially regulated by LC-PUFA is cyclooxygenase-2 (*cox2*), a key enzyme in prostanoid biosynthesis [29].

Recent studies conducted on broodstock diets of fish suggest that inclusion of VO as a major source of lipid may alter metabolic pathways in offspring of gilthead sea bream and improve the utilization of VM and VO by their offspring [30,31]. Therefore, higher levels up to 80% substitution of FO by VO in broodstock diets may upregulate *fads2* expression in offspring larvae and improve the utilization of low FM/FO diets at the juvenile stages of their life cycle [30]. This higher utilization of low FM/FO diets was persistent even in the 16-month-old offspring and affected the expression of some key gene encoding enzymes related to lipid utilization and LC-PUFA biosynthesis, including *lpl*, *cpt1*, *elovl6* [31]. More recently, it has also been demonstrated that broodstock fish showing high *fads2* expression levels in blood after one month feeding a low FO/high linseed oil (LO) diet had improved spawning performance, as well as growth of the offspring when nutritionally challenged with low FM/FO diets at the age of 6-months (Turkmen et al., submitted). However, increased dietary ALA, without a significant reduction in LC-PUFA, may lead to adverse effects on nutritional programming in offspring, resulting in lower growth as compared to offspring obtained from broodstock fed 100% FO. Such effects of ALA-rich broodstock diet or the selection of fish with a high *fads2* expression on offspring liver biochemical and fatty acid composition, or the potential molecular mechanisms involved, have not yet been studied.

Nutritional programming through parental diets has been well studied in humans, where early nutritional interventions during plastic developmental stages may alter the risk of cardiovascular diseases related to metabolic defects such as type 2 diabetes mellitus, hypertension, obesity, and osteoporosis [32]. Moreover, LC-PUFA supplementation during early nutrition can lead to long-term effects on metabolism by affecting the epigenome through different epigenetic mechanisms, including DNA methylation [33]. In murine models, maternal fat intake alters ARA (22:4*n-6*) and DHA contents in the liver, which was related to the epigenetic regulation of Fads2 gene promoter and the expression of the gene [34]. In fish, epigenetic studies are considered a relatively new area of research [35]. An earlier study showed that there was a negative correlation between the methylation status of the putative promoter region of the *fads2* and *fads2* gene expression in Japanese sea bass (*Lateolabrax japonicus*) [36]. However, in the European sea bass (*Dicentrarchus labrax*), the methylation level of several positions examined within the *fads2* promoter did not change after nutritional conditioning of larvae with high- or low-PUFA diets [37]. When these changes do occur, and whether the transcription of the corresponding gene and metabolic processes may be altered, remains to be established [33]. To our knowledge, there are no data regarding the epigenetic mechanisms involved in the nutritional programming effect of broodstock diets in gilthead sea bream. The recent publication of the whole genome for this species [38] and another on-going genome project, such as those of IATS-CSIC-Nutrigroup (http://nutrigroup-iats.org/seabreamdb/index.php), are opening new opportunities to understand potential epigenetic mechanisms.

The present study was designed to investigate the effects of parental *fads2* expression levels, and broodstock feeding a diet rich in VO, on their juvenile offspring response to a low FM and low FO diet. In particular, the changes in liver fatty acid composition, expression of genes involved in lipid metabolism and LC-PUFA biosynthesis, and the methylation status in a region of the *fads2* gene promoter were investigated.

## 2. Results

After one month of feeding the high VO and VM diet (Table 1), there were significant differences in the *fads2* expression in broodstock fish, which was up to 23 times higher in females (*p* = 0.01) and 13 times higher in males (*p* > 0.04) than the lowest *fads2* expression of each sex (Table 2). After dividing the broodstock fish by their *fads2* expression, the resultant high *fads2* expression group showed significantly higher expression than the low *fads2* group (*p* < 0.05) (Table 2). There was no link between fish weight and *fads2* expression of brood fish (*R* = 0.0061, *p* > 0.05).

**Figure 1:**
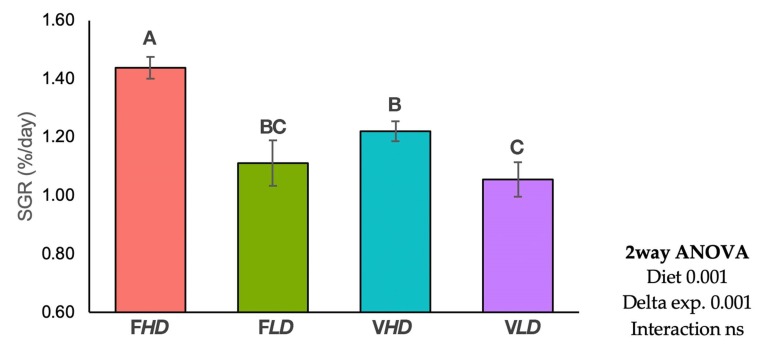
Specific growth rate (SGR, %/day) of progeny obtained from different broodstock groups after nutritional challenge test with very low fishmeal and fish oil diets. * Different letters denote significant differences among experimental groups (*p* < 0.05). ns means no significance difference (*p* > 0.05). Error bars shows the standard deviation (SD). Specific growth rate (SGR. %/day) = [Ln (final weight. g) – Ln (initial weight. g)]/(number of days) × 100.

The nutritional challenge of the juveniles with the very low amounts of FM and FO diet (5% FM and 3%FO) (Table 3) resulted in higher SGR values for the offspring of broodstock origin with a higher *fads2* expression (F*HD* and V*HD*), than for those coming from parents with a lower expression (F*LD* and V*LD*) (*p* < 0.001, two-way ANOVA, Figure 1), particularly, when parents were fed diet F (*p* < 0.05, Figure 1). Besides, offspring from broodstock fed the V diet showed lower growth than offspring from parents fed the F diet (*p* < 0.001, two-way ANOVA, Figure 1). In offspring obtained from broodstock fed V diet (V*HD* and V*LD*), those from broodstock with lower *fads2* expression (V*LD*) showed significantly higher HSI (*p* < 0.05, Table 4). In addition, offspring from broodstock with lower *fads2* expression (F*LD* and V*LD*), and fish fed diet V (V*LD*) had higher HSI (*p* < 0.05, Table 4). Thus, HSI was increased in offspring of parents with lower *fads2* expression (*p* < 0.05, two-way ANOVA), particularly when broodstock were fed diet V, showing an interaction between broodstock *fads2* expression and broodstock diets (*p* < 0.05, Table 4).

Crude protein, crude lipid and ash contents of liver were not significantly different among the experimental groups (*p* > 0.05, Table 4), despite a 6–13% increase in hepatic lipid content of juveniles from broodstock fish with lower *fads2* expression. However, offspring from broodstock with lower *fads2* expression (F*LD* and V*LD*) showed significantly higher monounsaturated fatty acids 16:1*n-5* and 20:1*n-5*, as well as some medium chain PUFAs such as 16:3*n-4*, 18:2*n-4* and, particularly, 18:2*n-6* (substrate for Fads2), or the LC-PUFAs 20:2*n-9*, 20:3*n-6*, 20:5*n-3*, 22:5*n-3*, or 22:6*n-3* (*p* < 0.05, Table 5).

The comparison of juveniles from broodstock fed the diet V (V*HD* and V*LD*) showed that in V*LD* juveniles, products of Fads2 activity such as 20:2*n-9* (*p* < 0.05) and 20:3*n-6* (*p* < 0.1) were reduced, or did not change, such as 18:2*n-9*, 18:3*n-6*, 18:4*n-3*, or 20:4*n-3* (*p* > 0.05), and Fads2 substrates such as 20:1*n-9*, 20:2*n-6*, 18:1*n-9*, 18:2*n-6*, 18:3*n-3*, or 20:3*n-3* were also not significantly affected. 

A reduction in a substrate for Elovl6, 16:0 (*p* = 0.06) was observed in F*LD* and V*LD* fish, but 18:1 product was similar among the experimental groups (*p* > 0.05) (Table 5). Broodstock diet had no significant (*p* > 0.05) effect on fatty acid profiles of the liver tissue of the offspring juveniles (Table 5), except for a trend towards lower values of saturated fatty acids F*LD* and V*LD* (Table 5, *p* > 0.05). No interaction between broodstock diet and *fads2* expression was observed on fatty acid profiles, except for the content in a minor fatty acid, 16:0ISO (*p* < 0.05, Table 5).

**Figure 2:**
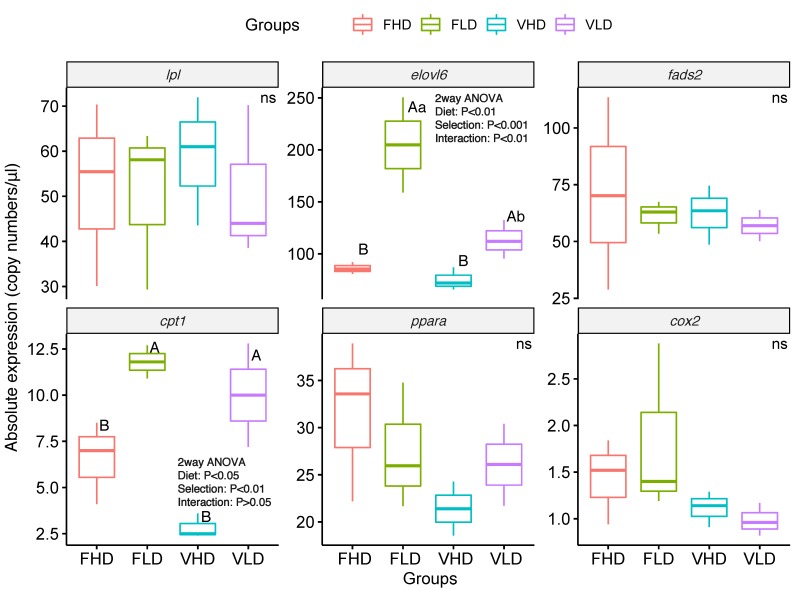
Box-and-whisker plots of absolute gene expression (copy numbers/μL) of six different genes after challenging 6-month-old gilthead seabream individuals with high vegetable oil and meal feeds for 2 months. Complete gene names; *lpl*: Lipoprotein lipase, *ppara*: Peroxisome proliferator-activated receptor alpha, *elovl6*: Elongation of very long chain fatty acids protein 6, *fads2*: Fatty acid desaturase 2, *cox2*: Cyclooxygenase-2, *cpt1*: Carnitine palmitoyltransferase I. Indications are as follows: *p*-values of two-way ANOVA analysis were shown inside the tables if *p*-values were < 0.05. Letters indicate differences between groups, ns means no significance difference (*p* > 0.05). *n* = 3 for all groups and genes.

In terms of the expression of selected genes, juveniles from parents with low *fads2* expression (F*LD* and V*LD*) showed a significantly (two-way ANOVA, *p* < 0.001, Figure 2) higher expression of *elovl6,* particularly when broodstock were fed with diet F. Thus, *elovl6* expression was approximately 2 times higher in F*LD* group in comparison with F*HD* (*p* < 0.05) (Figure 2), whereas between juveniles from parents fed V diet, the expression of this gene was only 1.3 times higher in V*LD* fish than in V*HD* group (*p* < 0.05) (Figure 2). 

Feeding broodstock with the diet V significantly (two-way ANOVA, *p* < 0.01, Figure 2) reduced *elovl6* expression in the offspring juveniles, particularly in those from broodstock with lower *fads2* expression. Thus, juveniles from lower *fads2* expression broodstock showed a significantly lower expression in *elovl6* when their parents were fed diet V (V*LD* juveniles) as compared to the parents were fed diet F (F*LD* juveniles, *p* < 0.05) (Figure 2). A significant interaction between broodstock *fads2* expression and broodstock diet on the expression of *elovl6* in their juveniles was observed (two-way ANOVA, *p* < 0.01, Figure 2). The expression of *cpt1* followed a similar trend, although the downregulation effect of broodstock diet V had lower significance (two-way ANOVA, *p* < 0.05, Figure 2). Thus, juveniles from parents with low *fads2* expression (F*LD* and V*LD*) showed a significantly (two-way ANOVA, *p* < 0.001, Figure 2) higher expression of *cpt1* than those from parents with higher expression (F*HD* and V*HD*). Despite selection of broodstock for low *fads2* expression and feeding diet V that resulted in downregulation of the expression of *fads2* and *cox2* in juveniles, there were no significant differences among juveniles in the expression of these two genes (*p* > 0.05, Figure 2). Neither there were differences in *lpl* or in *ppara* gene expressions (*p* > 0.05, Figure 2).

**Figure 3:**
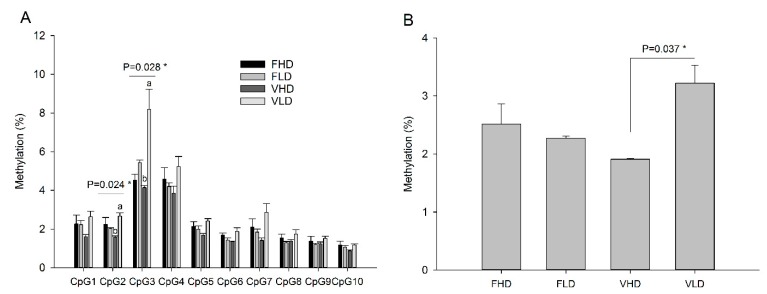
(**A**) Site-specific methylation level of 10 CpG positions in promoter region of the *fads2* gene, in livers of gilthead sea bream offspring juvenile obtained from selected broodstock fed with either 100% fish oil (F) or 30% FO – 70% VO (V) diets. (**B**) Average methylation of 10 CpG positions within a CG island in the promoter of *fads2*. * = *p* < 0.05 and different letters denote significant differences between treatments, no letters mean *p* > 0.05.

In general, a low level of cytosine methylation was found for the studied fragment of *fads2* promoter. Methylation level was always <10% for individual CpG positions and <4% for the average of all CpG positions examined (Figure 3). However, few differences were observed in individuals with CpGs; methylation at positions CpG2 and CpG3 in offspring juveniles from broodstock fed V diet was significantly higher when broodstock had a low *fads2* expression (V*LD*)(*p* < 0.05) (Figure 3a). Although not statistically significant, the same trend was observed for all other positions analysed and consequently, the average value for V*LD* juveniles was higher than for V*HD* juveniles (*p* < 0.05) (Figure 3b). Two-way ANOVA of methylation data for CpG2 and CpG3 revealed that in any case the parents’ diet had a significant effect (CpG2: *f* = 0.0006, *p* = 0.981; CpG3: *f* = 3.30, *p* = 0.099). The level of *fads2* gene expression of parents (i.e., selection) had no effect on the methylation level at CpG2 (*f* = 4.34, *p* = 0.058) while it had a significant effect at CpG3 (*f* = 14.13, *p* = 0.004). For both CpG positions, a significant interaction between parents’ selection and nutrition was found for methylation level (CpG2: *f* = 10.11, *p* = 0.007; CpG3: *f* = 5.75, *p* = 0.037).

## 3. Discussion

Prior to one month of spawning of broodstock fish fed a high VM and VO diet showed a wide variation in *fads2* expression in peripheral blood cells. Some females showed 23 times higher *fads2* expression than the lowest values found in other females. Whereas males had the highest *fads2* expression values, up to 13 times higher than the lowest values for each sex. This variation, and the higher expression in female individuals could be related to a potential higher requirement for DHA in females than in males, since this fatty acid plays an essential role in embryonic development [40]. Similar to this finding, female mammals maintain higher levels of DHA in liver and plasma phospholipids because of their ability to synthesise higher levels of DHA than male counterparts [41]. Moreover, studies in mouse have showed a very high correlation between reproductive hormones, such as progesterone and estradiol, and FADS2 expression as well as DHA concentration [42]. To date, the knowledge of the relationship between reproductive hormones and *fads2* gene expression in fish is scarce. Further studies are needed to clarify this relationship in gilthead sea bream.

After one month of feeding the low FM and low FO diet (5% FM and 3% FO), juvenile offspring obtained from broodstock selected with high *fads2* expression showed better growth and lower liver 18:2*n-6* concentration, a substrate for Fads2, than juveniles from broodstock with low *fads2* expression. Interestingly, juveniles obtained from broodstock with high *fads2* expression showed the largest variation in *fads2* expression in liver, as well as the highest values. However, there were no significant differences among fish groups in *fads2* gene expression, probably due to large variations among individual fish. It appears that the combined genetic and nutritional effects of parents on offspring growth resulted in higher growth of fish obtained from high *fads2* expression and parents fed diet F. Growth was higher in high *fads2* than low *fads2* groups regardless of parental diet intake. Studies in humans showed that LC-PUFA metabolism in babies could be affected by maternal FADS2 genetic and epigenetic status [43].

However, in the absence of a study on the heritability of *fads2* gene, it is difficult to interpret the reason for these individual differences. Ongoing studies with the known kinship of broodstock fish will reveal more information on the genetic variability and the parental effects of selection based on *fads2* gene expression. The putative epigenetic results are in agreement with previous studies on gilthead sea bream that showed the long-term effects of broodstock feeding diets with FO substitution by LO and a similar diet at the early stages of juvenile offspring. This may also explain part of the variation observed in *fads2* expression in the progeny originated from high *fads2* expression parents [31]. Nevertheless, it should be taken into account that the substitution levels of FO by LO were not significantly different between earlier and current studies (60% vs. 70%). However, Fads2 products (ARA, EPA, and DHA) were higher in fish fed V diets in this study, which may have led to lower variation in the progeny obtained from V diet fed groups.

Broodstock showing low *fads2* expression associated *elovl6* expression in the offspring is in agreement with lower 16:0 in the liver of juveniles with respect to those from broodstock with high *fads2* expression. Elovl6 is a key rate-limiting enzyme in long-chain fatty acid elongation and is involved in elongation of 16:0 and 16:1 to 18:0 and 18:1 fatty acids. Moreover, it has been shown that it is the sole enzyme with the ability to elongate 16:0, as shown in ELOVL−/− mice [44]. The content of 16:0 in liver decreased in fish obtained from low *fads2* groups (*p* = 0.06) and 16:1*n-5* increased (*p* < 0.04)(Table 5), in line with higher *elovl6* expression. However, 18:1 fatty acid showed no clear trend in relation with the *elovl6* expression, and this could be related to the relatively low levels of 18:0 (3.53%) and the high levels of 18:1*n-9* (15.25%) fatty acids in the juvenile diets.

In juveniles from low *fads2* groups, *cpt1* was also upregulated as compared to those from broodstock with high *fads2* expression. It is weidely recognized that Cpt1 is a key enzyme for energy production through the β-oxidation of fatty acids, which are transported into the mitochondria in the form of fatty acyl-carnitine esters by carnitine acyltransferases in fish [21]. Therefore, the upregulation of *cpt1* gene would imply an increase in liver β-oxidation, in agreement with a 6–13% reduction in hepatic lipid content of juveniles obtained from low *fads2* groups. A downregulation of both *elovl6* and *cpt1* in these groups were probably linked, since, Cpt1 gene expression in the liver of Elovl6 knock-out rats was also downregulated, leading to specific changes in the fatty acid ratios in liver [17] similar to those observed in this study. In addition, in the liver of Elovl6 knock-out rats, changes in chain length of fatty acids (decrease in LC-PUFA higher than 18C) and the ratio of fatty acids (C18:0/C16:0, C16:1/C16:0) reduced sterol regulatory element-binding protein 1 (Srebp-1) and Ppara [17]. PPARa also has an important role in energy metabolism, and its deficiency causes obesity in rats [45]. Thus, modulation of *elovl6* expression may be partly responsible for the improved growth in offspring of broodstock selected for high *fads2*. Moreover, when juveniles were obtained from broodstock fed the V diet, broodstock showing low *fads2* expression had the lowest growth and also increased HSI in comparison to juvenile from broodstock with high *fads2* expression. Besides, juveniles from broodstock with low *fads2* expression showed significantly increased methylation at CpG2 and CpG3 positions in the promotor region of *fads2*. These results suggest a lower ability of juveniles to transcribe *fads2* in comparison to those from broodstock with high *fads2* expression and fed the V diet, and requires further investigation. Liver fatty acid profiles of juveniles obtained from broodstock with low *fads2* expression also showed that products from Fads2 activity such as 20:2*n-9* and 20:3*n-6* were significantly reduced and showed no change in 18:2*n-9*, 18:3*n-6*, 18:4*n-3*, and 20:4*n-3* fatty acids as compared to juveniles from broodstock with high *fads2* expression and fed the V diet. Moreover, the substrates for Fads2 such as 20:1*n-9*, 20:2*n-6*, 18:1*n-9*, 18:3*n-3*, and 20:3*n-3* did not change, similar to 18:2*n-6*. Both of these fatty acid profiles and *fads2* expression, suggest again a lower Fads2 activity in juveniles of the low *fads2* expression parent origin, in comparison to juveniles from broodstock with high *fads2* expression and fed the V diet. Nevertheless, as discussed above, the large variation in the *fads2* expression values limited finding specific changes among juveniles of different origins. 

In recent years, fish epigenetics has been an emerging area of research interest from different scientific perspectives. However, studies on epigenetic mechanisms in most farmed fish are few, limited partly due to the limited molecular tools available for research and development. To the best of our knowledge, this is the first study describing the promoter methylation pattern of certain genes related to lipid metabolism (i.e., *fads2*) in gilthead sea bream. A previous study conducted on Japanese seabass showed that fish fed a *n-3* LC-PUFA rich diet showed higher methylation in the promoter of *fads2*, while those fed VO showed around 4% lower methylation levels [36]. The increased methylation of the promoter region of *fads2* led to lower gene expression of *fads2* in negative correlation to methylation levels. These findings agree with the higher methylation at certain positions of the *fads2* promoter in the liver, and the lower liver content of fatty acid products of Fads2 activity found in the progeny of broodstock fish selected for low *fads2* expression. However, in our study, the difference between the lowest (V*HD*) and the highest (V*LD*) of average methylation of 10 CpG positions only reached 1.5%, and this could contribute to the high variation in the *fads2* expression of the progeny obtained from high *fads2* expression broodstock that showed the lack of significant response of *fads2* expression. Studies in rats have showed differential expression of the Fads2 gene in negative correlation with its promoter methylation. However, the change in methylation pattern was higher (up to 15% change in the methylation on -623 region of the promoter) [34] in comparison to this study. Therefore, broodstock selection for low *fads2* expression, together with VO broodstock fed diets, only caused epigenetic changes at the *fads2* gene promoter, in the form of relatively small differences in cytosine methylation at certain positions. Indeed, increased promoter methylation of the *fads2* gene in offspring originated from low *fads2* groups, induced a small but statistically significant difference in the methylation of certain CpG positions within the *fads2* promoter that did not affect *fads2* expression levels. This result may suggest that the positions CpG2 and CpG3 are more likely to change in methylation due to their sequence context or other related factors. Since the parents diet did not affect the offspring methylation at these positions whereas the *fads2* expression of parents had a significant effect as well as its interaction with parents diet, it is posible that the selection of parents in this study resulted in the segregation of epialleles (i.e., a specific DNA methylation pattern of a locus) with different susceptibility to change methylation at these positions. Whether the above-mentioned and other CpG positions are involved in the transcriptional regulation of *fads2* under certain nutritional regimes or in particular genotypes remains to be elucidated. Expression of *fads2* among groups was similar, and the higher concentrations of EPA and DHA in offspring from broodstock with lower *fads2* expression and fed VO could be related to the selective retention of these fatty acids in the liver, rather than to *n-6* or *n-3* LC-PUFA synthesis. Other data from the same individuals showed that ARA, EPA, and DHA contents in other tissues—such as muscle content—were not affected (Turkmen et al., submitted). Further studies are needed to fully understand the relationship between *fads2* gene promoter methylation and corresponding gene expression.

Feeding broodstock with diet V produced juveniles with reduced *elovl6* expression in the liver, particularly broodstock with a low *fads2* expression. These results were in agreement with the 15–21% reduction in hepatic lipid contents and a trend towards lower saturated fatty acid content. In addition, juveniles from broodstock fed the V diet also showed a reduction in *cpt1* expression. These results are in agreement with the previous study that showed downregulation of *cpt1* in the liver of progenies from broodstock fed increased contents of dietary VO [31]. However a negative correlation was found between the level of VO inclusion in the broodstock diets and *cpt1* expression in the offspring juveniles when fed low FM and low FO diets in this report.

Likewise, feeding broodstock with the ALA rich diet (16.3% ALA of the total fatty acid profile, V diet) produced juveniles with a reduced growth when challenged with the low FM and FO diet. In summary, these results indicate a nutritional programming effect of FO replacement by ALA, when the products of Fads2 (ARA, EPA, and DHA) were similar in the eggs [LC-PUFA (% of total fatty acids), F diet: 20.29±2.70 and 18.44±4.02]. In previous studies, feeding gilthead sea bream broodstock a diet with 60% FO replacement by LO increased the ALA content in the eggs (13.08% of the total fatty acids) but reduced products of Δ-6-desaturase enzyme (ARA, EPA, and DHA). Modification of these fatty acids in the eggs generated a nutritional programming effect by producing juveniles that grew better when fed low FM and low FO diets. However, higher (80% and 100%) levels of FO replacement in broodstock diets negatively affected juveniles growth [30,31]. In these studies, 60% replacement of FO by VO also resulted in an increase in the amounts of LC-PUFA precursors such as LA and ALA up to 0.5 and 4.8 times in eggs, but it was accompanied by a decrease in products of *n-3* LC-PUFA biosynthesis such as EPA and DHA (0.3 and 0.2 times, respectively) [30]. However, in the present study, due to the contribution of the *n-3* LC-PUFA fatty acids from the FM source used, the dietary levels of EPA and DHA changed to a lesser extent (0.01 and 0.06 times, respectively) between both diets used in the nutritional programming, while differences between LA and ALA were 0.03 and 2.6 times, respectively. In comparison to our previous studies, [30,31,46], it appears that an increase in precursors, but not a reduction in products of *n-3* LC-PUFA synthesis, resulted in a programming effect as evidenced by a reduction in the juvenile growth when challenged with low FM/FO diets. There are few studies on the effects of the increase in the levels of LA and/or total PUFA intake on the inhibition of the PUFA metabolic pathway. It has been shown that high intake of 18C fatty acids and very low *n-3* LC-PUFA levels might inhibit *fads2* expression in gilthead sea bream [15,30]. Moreover, studies on Atlantic salmon have shown that *n-3* LC-PUFA and DHA, but not EPA, were responsible for the downregulation of *fads2* and elovl2-like elongase in liver [47]. In addition, the ratio of ALA to LA is important because it determines the DHA accumulation in plasma phospholipids [48]. If the ratio of LA to ALA is low (0.5–0.8), as in diet V of this study, increasing levels of ALA to 2% of dietary energy content increases plasma DHA phospholipid, however, DHA levels declined sharply when ALA was above 6% of total energy content [48].

## 4. Materials and Methods 

All the mentioned experiments below were conducted according to the European Union Directive (2010/63/EU) on the protection of animals for scientific purposes, at Fundación Canaria Parque Científico Tecnológico (FCPCT), University of Las Palmas de Gran Canaria (Canary Islands, Spain). All the experimental conditions and sampling protocols have been approved by the Animal Welfare and Bioethical Committee from the University of Las Palmas de Gran Canaria, December 2016.

### 4.1. Fish Feeding 

**Figure 4:**
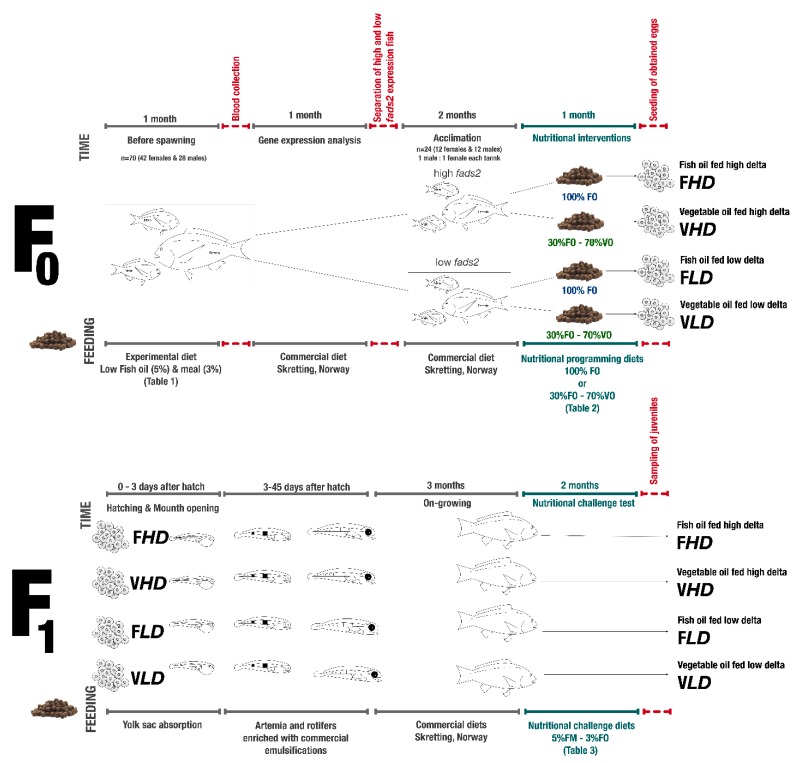
Experimental design and sampling points, for the simplicity only one replicate shown in the figure, in the experiment all groups were tested in triplicates. Sampling points were shown as 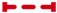, and experimental periods when fish were fed with different diets are shown as 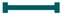.

The research conducted in this investigation had three phases. In the first phase, broodstock were separated on the basis of their *fads2* expression. In the second phase, each broodstock was fed with either a VO diet to induce nutritional programming or a control diet. In the last phase, the progeny obtained from these groups were fed a diet low in FM (5%) and FO (3%) at their juvenile stage (Figure 4).

In the first phase of the study, prior to the beginning of the spawning period, 70 gilthead sea bream broodstock fish, including 42 females and 28 males, were fed a high VO and VM diet (Table 1) to trigger *fads2* expression and for the identification of broodstock individuals with high (*HD*) or low expression values (*LD*). Fish were fed at a 1% biomass ratio at 08:00 and 14:00 h daily, except Sundays, for one month. After this period, fish were anaesthetized with 10 ppm clove oil/methanol (1:1 v/v) in seawater, and 2 mL blood samples were taken from the caudal vein with 2 ml sterile syringes (Terumo Europe NV, Leuven, Belgium). Blood was collected in 2.0 mL K 3 EDTA tubes (L.P. Italiana, Milan, Italy), and after mixing 1 ml of non-coagulated blood was transferred to 2 ml Eppendorf tubes. Blood samples were kept on ice during sampling and immediately centrifuged at 3000 rpm, 4 °C, for 10 min. Plasma was separated, and blood cells were quickly frozen with liquid nitrogen and kept at −80 °C until molecular studies were conducted. Broodstock showing the highest or the lowest *fads2* expression in blood were separated into two groups, *HD* and *LD*, respectively (Table 2).

In the second phase of study involving nutritional programming for the offspring, 12 brood fish of each sex from the high- (*HD*) and 12 from the low- (*LD*) *fads2* expression group, with similar length and weight (*p* > 0.05, Table 2), were distributed into 12 experimental tanks in a flow-through system with filtered seawater (37.0 ± 0.5‰ salinity, 19.59–21.30 °C) at a renewal rate of 100% per hour with proper aeration.

Substitution of FO by LO in the broodstock diet V increased the 18C fatty acids contents such as LA and ALA, which are substrates for Fads2, with levels 1.8 and 18.1 times higher in V than in F diet respectively. Whereas, with 20 and 22C fatty acids, the end products of fatty acid desaturation and elongation—such as ARA, EPA, and DHA—were the same (Table 6). Broodstock groups were fed twice a day (08:00 and 15:00 h) with a daily ration based on 1% initial body weight for each of the experimental diets for 37 days. At the end of this feeding period, eggs were collected from all groups and offspring were reared on the same commercial diets. Larvae were reared under natural photoperiod and living phytoplankton (*Nannochloropsis sp.;* 250 ± 100 × 10^3^ cells per mL) was added to the rearing tanks. From 3–17 dah, larvae were fed twice a day with rotifers (*Brachionus plicatilis*) at 10 rotifer/mL enriched with commercial emulsions (ORI-GREEN, Skretting, Norway). From 15–32 dah, *Artemia sp.* enriched with commercial emulsions (ORI-GREEN, Skretting, Norway) was added to the rearing tanks 3 times a day. In addition to the live feeds, from 20 dah, larvae were fed commercial diets according to the suggested diet particle size by the manufacturer until larvae reached 45 dah (Skretting, Norway). Fishes were reared using the same commercial diets (Skretting, Norway) for all the experimental groups until they reach desired juvenile size for the feeding challenge experiment. In the third phase of the study, the offspring from these four broodstock groups (F*HD*, F*LD*, V*HD*, and V*LD*) at their juvenile stage were nutritionally challenged with a low FM and low FO diet (5%FM and 3%FO, Table 3), in order to determine the effect of broodstock with differential *fads2* expression and ability of the offspring to utilize low FM and FO diets. There was no significant difference (*p* > 0.05) in the initial weights of the six-month-old juveniles at the beginning of the nutritional challenge test (F*HD*: 23.4±0.1, F*LD*: 22.1±0.1, V*HD*: 23.8±0.4, and V*LD*: 23.7±0.1). Juvenile fish final weights at the end of the experimental period were FHD: 47.4±0.8, FLD: 38.1±1.3, VHD: 43.3±0.1 and VLD: 37.8±1.2.

Six-month-old juvenile offspring from each of the broodstock groups were distributed into twelve 500 L tanks and fed the diets used for nutrtional challenge (Table 3) twice a day except Sundays at 08:00 and 14:00 h for 60 days. Average water temperature and pH during the trial was 22.8 ± 1.2 °C and 8.2 ± 0.3, respectively. Feed consumption was recorded daily. Feed was withdrawn for 24 h prior to growth measurements. 

Prior to all measurements and samplings, all fish (*n* = 75 per treatment) were anaesthetized with 10 ppm clove oil/methanol (1:1 v/v) in seawater. Specific growth rate (SGR) values were calculated using: [Ln (final weight. g) – Ln (initial weight. g)]/(number of days) × 100. 

### 4.2. Molecular Studies

#### 4.2.1. Quantification of fads2 Expression of Brood Fish

Blood was collected from 70 fish for the identification of *fads2* expression. In each Eppendorf tube (2 mL), 200 µL of blood cells was transferred and 1 mL of TRI Reagent (Sigma-Aldrich, San Luis, MO, USA) was added. Four pieces of 1 mm diameter zirconium glass beads were added to each tube, and the sample homogenized in a TissueLyzer-II (Qiagen, Hilden, Germany) for 60 s at a frequency of 30/s. Homogenates were then diluted with 250 µL chloroform and centrifuged at 12000 G for 15 min at 4 °C. The clear upper aqueous phase containing RNA was mixed with 75% ethanol and transferred into an RNeasy spin column where total RNA bonded to the membrane. After that, RNA was extracted using Qiagen RNeasy Mini Kit (Qiagen, Hilden, Germany) and the protocol supplied by the manufacturer. Quantity and quality of RNA were assessed by NanoDrop 2000c spectrophotometer (Thermo Scientific, Waltham, MA, USA) and integrity was analyzed in 1.5% agarose gels. Real-time quantitative PCR was performed in an iQ5 multi-colour real-time PCR detection system (Bio-Rad, Hercules, CA, USA) using beta-actin (*actb*) as the housekeeping gene in a final volume of 15 μL/reaction well, and with 100 ng of total RNA reverse-transcribed to complementary DNA (cDNA). Housekeeping gene, cDNA templates and reaction blanks were analysed in duplicate. Primer efficiency was tested with serial dilutions (1:5, 1:10, 1:100 and 1:1000) of a cDNA pool. Sequences of the primers used in this study for real-time quantitative PCR analysis of expression were: *actb* (GeneBank access no: KY388508) 5’–3’ (F): TCT GTC TGG ATC GGA GGC TC, 5’–3’ (R): AAG CAT TTG CGG TGG ACG, *fads2* (GeneBank access no: GQ162822) 5’–3’ (F): GCA GAG CCA CAG CAG CAG GGA, 5’–3’ (R): CGG CCT GCG CCT GAG CAG TT. Ninety-six-well PCR plate was used to analyze each gene, primer efficiency, and blanks. Melting-curve analysis was performed to confirm the amplification of a single product after each run. Fold expression of each gene was determined by 2ˆ(-delta-delta CT) method (2^−ΔΔ*C*t^) [49]. PCR efficiencies were similar, and no efficiency correction was required [49,50]. 

#### 4.2.2. Digital Droplet PCR Analysis for Absolute Gene Expression in Juveniles 

RNA extraction from the liver of juveniles (*n* = 9 from each experimental group) was carried out using a similar protocol described above for the qPCR. Absolute gene expression analysis was performed with Droplet Digital PCR (ddPCR) using Bio-rad QX200 (Hercules, CA, USA) systems, using the same cDNA obtained as above. Samples were prepared using the workflow provided by the manufacturer. In summary, for each gene, master mixes were prepared using 10 μL EvaGreen super mix (Bio-rad, Hercules, CA, USA), 0.2 μL F primer, 0.2 μL R primer, 7.6 μL MilQ water, and 2 μL cDNA (approx. 20 ng cDNA). Primers, Genbank accession numbers and reference articles for sequences of target (*lpl*, *ppara*, *elovl6*, *fads2*, *cox2*, *cpt1*) and housekeeping gene (*ß-act*) were provided in Supplementary Table S1. Droplets were generated using droplet generator Bio-rad QX200 (Hercules, CA, USA) and were transferred to 96-well microplates for PCR in a thermal cycler (Bio-rad C1000 Touch, Hercules, CA, USA). Following PCR amplification, droplets were read in a droplet reader (Bio-rad QX200, Hercules, CA, USA) to determine absolute gene expression. Readings lower than 12000 droplets were not used for the gene expression.

#### 4.2.3. DNA Methylation Analysis

DNA was extracted from liver samples of juvenile fish using the Quick-DNA™ Miniprep Plus Kit (Zymo Research, Irvine, CA, USA) following the manufacturer’s instructions. Quantity and quality of DNA were assessed by NanoDrop 2000c Spectrophotometer (Thermo Scientific, Waltham, MA, USA) and integrity was analyzed in 2% agarose gels. Samples were stored at −20 °C until further processing. DNA was converted to bisulfite using the EZ DNA Methylation Gold (Zymo Research, Irvine, CA, USA) bisulfite conversion kit following the manufacturer’s recommendations. Primers were designed using the PyroMark assay design software (version 2.0.01.15, Hilden, Germany) to hybridize CpG-free sites and to have the highest possible Tm. Reverse primers were labeled with biotin at the 5’-end to allow the capture of the biotinylated strand of the amplified DNA for further pyrosequencing. The target sequence was located within a previously identified CpG island at the *fads2* gene promoter, as determined by MethPrimer (http://www.urogene.org/methprimer/), and it contains 10 CpG sites. Search parameters for CpG islands were: length ≥ 200, C+G content ≥ 50%, the ratio of observed /expected CpGs ≥ 0.60 and window size= 100. Bisulfite-converted DNA was amplified by PCR, using the Invitrogen™ Platinum™ Taq Hot-Start DNA Polymerase (Thermo Fisher Scientific, Waltham, MA, USA) and forward- and reverse-primers at 1 µM each in a total volume of 25 µL. Two regions were examined and were referred as ’left’ and ’right’ according to their relative position 5’ to 3’ within the identified CpG island. Each region was amplified by a forward (F) and a biotinylated reverse primer (R), and pyro-sequenced with a sequencing primer (Seq) as follows: 

Left F: GTTGTAATTGAGGGAAAGTGTAGAA, 

Left R: [btn]CACCCACTCATTCAATACAAATTC, 

Left Seq: AGGGAAAGTGTAGAAG; 

Right F: GTTGTAATTGAGGGAAAGTGTAGAAG, 

Right R: [btn]TCATTCACCCACTCATTCAATACAAATTC, 

Right Seq: GGTGGTTTAGGATATATTG

Reactions were performed in an Eppendorf Mastercycler Ep Realplex (Eppendorf, Hamburg, Germany) with the following cycling: 95 °C for 5min, followed by 35 cycles of 95 °C for 45 s, 60 °C for 45 s, and 72 °C for 1.5 min and a final extension at 72 °C for 5min. PCR products were checked by 1% agarose gels to ensure specificity before pyrosequencing. Pyrosequencing reactions were carried out using the PyroMark Q24 instrument (Qiagen, Hilden, Germany) and data analysis was done using the PyroMark Q24 software. Only pyrosequencing reactions that passed the quality test were included in the analysis. Methylation values were expressed per position as the average of replicates, and as average methylation for the fragment as the mean of values across all sites analyzed. Sample size in the pyrosequencing assay for the different groups was F*HD* (*n* = 4), F*LD* (*n* = 5), V*HD* (*n* = 3), and V*LD* (*n* = 5). In each line, controls to assess pyrosequencing quality were included.

### 4.3. Statistical Analysis

The results were expressed as mean ± standard deviation (*n* = 3) if not otherwise stated in material methods section, tables, and figures. Data were compared statistically using analysis of variance (ANOVA) at a significant level of 5% to determine the effects of broodstock with different *fads2* expression capacity and/or feeding during the spawning period on the offspring. All residuals were checked for normality and homogeneity of variance using the Kolmogorov–Smirnoff and the Levene tests, respectively [51]. If significant differences were detected with ANOVA, means were compared by the Tukey test. All data were analyzed using IBM SPSS v23.0.0.2 for Mac (IBM SPSS Inc. Chicago, IL, USA). Pearson correlation test was performed to identify relationship between parameters using R (R Foundation for Statistical Computing, Vienna, Austria).

## 5. Conclusions and Future Perspectives

The aim of this investigation was to assess the combined effects of broodstock with different *fads2* expression levels and lipid nutrition during the spawning period on lipid metabolism-related genes in the liver—as well as to examine the effects of nutritional programming during the spawning season on DNA methylation of the *fads2* promoter region in gilthead sea bream offspring. Selection of gilthead sea bream broodstock with high fads2 expression when fed a low FM and FO diet produced offspring that performed better, even when they were challenged with a low FM and FO diet. Besides, selection of high *fads2* broodstock induced regulation of genes such as *elovl6* and *cpt1* in juveniles challenged with a very low FM FO diet. Certain key genes for lipid biosynthesis in liver, such as *elovl6* and *ppara*, were downregulated in offspring obtained from broodstock showing low *fads2* expression, while the broodstock diet had a limited effect on the expression of analyzed genes. The results of this study showed that methylation of CpG islands in the *fads2* promotor, particularly in positions CpG2 and CpG3, are among the potential epigenetic mechanisms for regulation of gene expression in nutritionally programmed fish. Studies are under way on the effects of genetic selection of broodstock by *fads2* expression levels, the improvement of nutritional programming diets, and epigenetic effects on the progeny.

## Figures and Tables

**Table 1:** Main ingredient component*, energy, protein, and % total fatty acids contents of diets used in the conditioning study

Main Ingredients (%)	%	Proximate Composition	(% Dry Matter)
Fish meal SA ^1^ 68 super prime ^1^	5.00	Crude lipids	21.7
Fish meal alternative protein sources ^2^	54.50	Crude protein	45.1
Rapeseed meal cake	11.30	Moisture	9.0
Wheat	6.89	Ash	5.4
Fish oil SA ^1^	3.00		
Vegetable oil mix ^3^	13.00	**Gross Energy (MJ/kg, as is)**	22.5
**% Total Fatty Acids**		**% Total Fatty Acids**	
14:0	6.6	18:3*n-3*	11.8
14:1*n-5*	0.1	18:4*n-3*	0.4
15:0	0.1	18:4*n-1*	0.0
16:0ISO	0.0	20:0	0.4
16:0	12.3	20:1*n-9*	0.0
16:1*n-7*	2.1	20:1*n-7*	1.0
16:1*n-5*	0.1	20:1*n-5*	0.1
16:2*n-4*	0.2	20:2*n-9*	0.0
17:0	0.3	20:2*n-6*	0.1
16:3*n-4*	0.1	20:3*n-9*	0.0
16:3*n-3*	0.0	20:3*n-6*	0.0
16:3*n-1*	0.0	20:4*n-6*	0.2
16:4*n-3*	0.4	20:3*n-3*	0.0
18:0	3.2	20:4*n-3*	0.1
18:1*n-9*	32.3	20:5*n-3*	2.5
18:1*n-7*	2.3	22:1*n-1*1	0.1
18:1*n-5*	0.0	22:1*n-9*	0.3
18:2*n-9*	0.0	22:4*n-6*	0.0
18:2*n-6*	20.3	22:5*n-6*	0.1
18:2*n-4*	0.1	22:5*n-3*	0.3
18:3*n-6*	0.1	22:6*n-3*	1.7
18:3*n-4*	0.0		

* Please see [39] diet code 5FM/3FO for the complete list of feed ingredients. ^1^ South American, Superprime (Feed Service, Bremen, Germany). ^2^ Blood meal spray (Daka, Denmark), soya protein concentrates 60% (Svane Shipping, Denmark), corn gluten 60 (Cargill, Netherlands), wheat gluten (Cargill, Netherlands). ^3^ Linseed (2.6%) (Ch. Daudruy, France), rapeseed (5.2%) (Emmelev, Denmark), and palm oils (5.2%) (Cargill, Netherlands).

**Table 2:** Body weight, length, and *fads2* expression levels of the various broodstock groups

Groups			F*HD*	F*LD*	V*HD*	V*LD*	ANOVA *
Weight	(kg)	♀	2.40 ± 0.33	1.99 ± 0.28	2.61 ± 1.19	1.84 ± 0.61	n.s.
		♂	2.05 ± 0.89	1.68 ± 0.13	1.30 ± 0.45	1.65 ± 0.48	n.s.
Lenght	(cm)	♀	45.75 ± 0.35	42.83 ± 1.44	45.33 ± 5.50	41.83 ± 3.75	n.s.
		♂	45.50 ± 1.41	39.83 ± 1.15	36.66 ± 2.02	40.83 ± 5.05	n.s.
*fads2* expression	(fold change)	♀	5.77 ± 2.41^a^	0.17 ± 0.18^b^	5.29 ± 1.91^a^	0.35 ± 0.08^b^	0.01
		♂	3.05 ± 1.11^a^	0.26 ± 0.06^b^	6.06 ± 6.26^a^	0.72 ± 0.13^b^	0.04

* Values in the same row with different letters indicate differences between groups (*n* = 3, *p* < 0.05). n.s. means no significance difference (*p* > 0.05). In the case of similar values, no indications were made (*p* > 0.05).

**Table 3:** Formulation, main ingredients, and biochemical composition of the nutritional challenge diets

Main Ingredients (%)	%	Proximate Composition	(% Dry Matter)
Fish meal SA ^1^ 68 super prime	5.0	Crude lipids	21.8
Fish meal alternative protein sources ^2^	54.5	Crude protein	57.2
Rapeseed meal cake	11.3	Moisture	6.5
Wheat	6.9	Ash	6.7
Fish oil SA ^1^	3.0		
Vegetable oil mix ^3^	13.0		
Micronutrient mixes ^4^	6.3	**Gross Energy (MJ/kg, as is)**	22.5
**% Total Fatty Acids**		**% Total Fatty Acids**	
14:0	4.69	18:3*n-4*	0.04
14:1*n-5*	0.17	18:3*n-3*	14.86
15:0	0.28	18:4*n-3*	0.91
16:0	13.67	20:0	0.21
16:1*n-7*	4.50	20:1*n-9*	0.37
16:1*n-5*	0.09	20:1*n-7*	7.25
16:2*n-6*	0.25	20:1*n-5*	0.37
17:0	0.13	20:2*n-6*	0.16
16:3*n-4*	0.14	20:4*n-6*	0.31
16:3*n-3*	0.14	20:3*n-3*	0.09
16:3*n-1*	0.04	20:4*n-3*	0.19
16:4*n-3*	0.22	20:5*n-3*	3.81
18:0	3.23	22:1*n-1*1	9.35
18:1*n-9*	15.75	22:1*n-9*	1.00
18:1*n-7*	2.46	22:4*n-6*	0.04
18:1*n-5*	0.23	22:5*n-6*	0.03
18:2*n-6*	9.84	22:5*n-3*	0.28
18:2*n-4*	0.05	22:6*n-3*	4.49
18:3*n-6*	0.08		

Please see [39] diet code 5FM/3FO for the complete list of feed ingredients. ^1^ South American, Superprime (Feed Service, Bremen, Germany). ^2^ Blood meal spray (Daka, Denmark), soya protein concentrates 60% (Svane Shipping, Denmark), corn gluten 60 (Cargill, Netherlands), wheat gluten (Cargill, Netherlands). ^3^ Linseed (2.6%) (Ch. Daudruy, France), rapeseed (5.2%) (Emmelev, Denmark) and palm oils (5.2%) (Cargill, Netherlands). ^4^ Micronutrient mix: Vitamin mix (0.75%); Supplied the following vitamins (mg/kg): A 3.8, D 0.05, E 102.4, K3 9.8, B1 2.7, B2 8.3, B6 4.8, B12 0.25, B3 24.8, B5 17.2, folic acid 2.8, H 0.14, C 80; minerals (mg/kg): cobalt 0.94, iodine 0.7, selenium 0.2, iron 32.6. manganese 12, copper 3.2, zinc 67; other (g/kg): taurine 2.45, methionine 0.5, histidine 1.36, cholesterol 1.13. DSM, (Netherlands), Evonik Industries (Germany), Deutsche Lanolin Gesellschaft (Germany), supplemented ingredients (5.49%); Contains lysine, methionine, monocalcium phosphate, choline, inositol, phospholipids, Vilomix (Denmark), Evonik Industries (Germany), Pöhner (Germany). Antioxidant (0.5%): BAROX BECP, Ethoxyquin, Vilomix (Denmark). Yttrium oxide (0.3%).

**Table 4:** Liver biochemical composition and hepatosomatic index of juvenile fish after the nutritional challenge test with a low fish meal and fish oil diets fed for 2 months

	Groups				Two-Way ANOVA *		
Composition (%)	F*HD*	F*LD*	V*HD*	V*LD*	Broodstock Diet (D)	Broodstock *fads2* Expression (*f*)	D × *f*
Moisture	64.2 ± 2.0	66.6 ± 5.9	66.5 ± 0.1	66.6 ± 4.2	n.s.	n.s.	n.s.
Protein	11.1 ± 0.3	10.1 ± 2.4	12.1 ± 0.4	11.2 ± 1.3	n.s.	n.s.	n.s.
Lipids	13.9 ± 2.1	14.1 ± 4.9	12.1 ± 1.8	11.0 ± 2.1	n.s.	n.s.	n.s.
Ash	2.5 ± 0.5	2.7 ± 1.2	2.9 ± 0.3	2.9 ± 0.4	n.s.	n.s.	n.s.
HSI ^†^	1.4 ± 0.2	1.2 ± 0.2 ^B^	1.3 ± 0.1 ^b^	1.6 ± 0.1 ^Aa^	n.s.	0.05	0.03

* Values in the same row have different letter indicates differences between groups (*n* = 3, *p* < 0.05). In case of similar values, no indications were made (*p* > 0.05). ns means no significance difference (*p* > 0.05). ^†^ Hepatosomatic index (HSI) = (liver weight/fish weight) × 100.

**Table 5:** Fatty acid composition of livers after challenge test diets with substitution of FO by LO (% total fatty acids)

	F*HD*	F*LD*	V*HD*	V*LD*	Diet (D)	*fads2*(*f*)	D × *f*
14:0	1.58 ± 0.09	1.66 ± 0.40	1.76 ± 0.27	1.58 ± 0.28	0.76	0.76	0.47
14:1*n-7*	0.01 ± 0.00	0.01 ± 0.00	0.02 ± 0.00	0.02 ± 0.00	0.14	0.69	0.52
14:1*n-5*	0.02 ± 0.01	0.04 ± 0.01	0.03 ± 0.00	0.03 ± 0.00	0.80	0.20	0.16
15:0	0.13 ± 0.01	0.17 ± 0.04	0.14 ± 0.02	0.14 ± 0.00	0.68	0.12	0.20
15:1*n-5*	0.02 ± 0.00	0.02 ± 0.00	0.02 ± 0.01	0.02 ± 0.00	0.88	0.51	0.11
16:0ISO	0.02 ± 0.00B	0.02 ± 0.00A	0.02 ± 0.00AB	0.02 ± 0.00B	0.49	0.43	**0.03**
16:0	15.70 ± 0.42	14.30 ± 0.96	16.02 ± 1.68	13.92 ± 1.92	0.97	0.06	0.67
16:1*n-7*	2.26 ± 0.17	2.49 ± 0.44	2.57 ± 0.23	2.43 ± 0.23	0.48	0.79	0.31
16:1*n-5*	0.11 ± 0.00B	0.13 ± 0.01A	0.12 ± 0.00B	0.14 ± 0.02A	0.24	**0.04**	0.38
16:2*n-4*	0.05 ± 0.02	0.09 ± 0.05	0.07 ± 0.02	0.08 ± 0.01	0.71	0.11	0.37
17:0	0.07 ± 0.01	0.11 ± 0.04	0.09 ± 0.02	0.10 ± 0.01	0.52	0.09	0.27
16:3*n-4*	0.19 ± 0.01B	0.21 ± 0.00A	0.19 ± 0.00B	0.20 ± 0.00A	0.80	**0.00**	0.07
16:3*n-3*	0.03 ± 0.00	0.04 ± 0.01	0.04 ± 0.00	0.04 ± 0.01	0.67	0.08	0.08
16:3*n-1*	0.01 ± 0.00	0.01 ± 0.00	0.01 ± 0.00	0.01 ± 0.00	0.11	0.50	0.19
16:4*n-3*	0.03 ± 0.01	0.05 ± 0.04	0.04 ± 0.02	0.05 ± 0.01	0.63	0.17	0.46
18:0	6.16 ± 0.54A	5.38 ± 0.17B	5.86 ± 0.37AB	5.40 ± 0.49B	0.57	**0.03**	0.52
18:1*n-9*	42.01 ± 0.34	40.03 ± 2.18	40.50 ± 0.48	40.05 ± 0.88	0.32	0.12	0.30
18:1*n-7*	2.52 ± 0.05	2.60 ± 0.14	2.54 ± 0.07	2.56 ± 0.04	0.86	0.31	0.54
18:1*n-5*	0.06 ± 0.01	0.08 ± 0.00	0.06 ± 0.03	0.07 ± 0.00	0.31	0.17	0.84
18:2*n-9*	2.04 ± 0.10	2.29 ± 0.49	2.26 ± 0.33	2.69 ± 0.88	0.35	0.30	0.78
18:2*n-6*	11.09 ± 0.41B	11.85 ± 0.33A	10.64 ± 0.56B	11.67 ± 0.63A	0.31	**0.01**	0.66
18:2*n-4*	0.06 ± 0.01B	0.09 ± 0.02A	0.07 ± 0.00AB	0.08 ± 0.01AB	0.85	**0.01**	0.26
18:3*n-6*	1.69 ± 0.04	2.17 ± 0.39	1.89 ± 0.46	2.49 ± 0.85	0.41	0.11	0.85
18:3*n-4*	0.06 ± 0.00	0.07 ± 0.01	0.07 ± 0.01	0.07 ± 0.00	0.32	0.20	0.11
18:3*n-3*	5.30 ± 0.24	5.67 ± 0.44	5.15 ± 0.57	5.74 ± 0.45	0.89	0.10	0.68
18:4*n-3*	1.21 ± 0.01	1.60 ± 0.23	1.41 ± 0.39	1.80 ± 0.53	0.36	0.09	1.00
18:4*n-1*	0.02 ± 0.00	0.04 ± 0.01	0.03 ± 0.00	0.04 ± 0.00	0.32	0.10	0.19
20:0	0.16 ± 0.00	0.15 ± 0.03	0.16 ± 0.02	0.14 ± 0.02	0.69	0.41	0.87
20:1*n-9*	0.12 ± 0.01	0.14 ± 0.02	0.12 ± 0.01	0.13 ± 0.01	0.51	0.19	0.69
20:1*n-7*	0.73 ± 0.05	0.71 ± 0.18	0.74 ± 0.11	0.66 ± 0.09	0.83	0.48	0.69
20:1*n-5*	0.07 ± 0.00B	0.08 ± 0.01AB	0.07 ± 0.01B	0.08 ± 0.00A	0.49	**0.04**	0.45
20:2*n-9*	0.75 ± 0.12AB	0.51 ± 0.12BC	0.88 ± 0.02A	0.47 ± 0.14C	0.52	**0.00**	0.23
20:2*n-6*	0.26 ± 0.02	0.25 ± 0.02	0.24 ± 0.01	0.23 ± 0.02	0.11	0.23	0.92
20:3*n-9*	0.00 ± 0.00	0.01 ± 0.00	0.00 ± 0.00	0.01 ± 0.00	0.71	0.12	0.48
20:3*n-6*	0.27 ± 0.03A	0.22 ± 0.03B	0.31 ± 0.02A	0.21 ± 0.04B	0.37	**0.00**	0.19
20:4*n-6*	0.35 ± 0.00	0.39 ± 0.05	0.35 ± 0.02	0.37 ± 0.02	0.57	0.17	0.50
20:3*n-3*	0.23 ± 0.02	0.22 ± 0.01	0.21 ± 0.01	0.21 ± 0.01	0.07	0.46	0.67
20:4*n-3*	0.30 ± 0.03	0.32 ± 0.04	0.38 ± 0.04	0.33 ± 0.01	0.07	0.39	0.12
20:5*n-3*	0.96 ± 0.12B	1.42 ± 0.38AB	1.21 ± 0.10AB	1.42 ± 0.16A	0.37	**0.03**	0.36
22:1*n-1*	0.17 ± 0.04	0.24 ± 0.12	0.21 ± 0.06	0.22 ± 0.02	0.87	0.35	0.53
22:1*n-9*	0.27 ± 0.01	0.26 ± 0.04	0.26 ± 0.04	0.24 ± 0.02	0.37	0.35	0.57
22:4*n-6*	0.04 ± 0.00	0.05 ± 0.01	0.05 ± 0.01	0.05 ± 0.00	0.83	0.15	0.36
22:5*n-6*	0.10 ± 0.00	0.12 ± 0.01	0.10 ± 0.01	0.11 ± 0.01	0.90	0.05	0.13
22:5*n-3*	0.39 ± 0.05B	0.61 ± 0.12A	0.49 ± 0.04AB	0.61 ± 0.09A	0.35	**0.01**	0.37
22:6*n-3*	2.37 ± 0.16B	3.09 ± 0.51AB	2.60 ± 0.18B	3.10 ± 0.38A	0.56	**0.01**	0.57
**Σ**							
Saturated^1^	23.80 ± 0.84	21.77 ± 1.59	23.03 ± 1.78	21.29 ± 2.58	0.57	0.11	0.89
16:1n^2^	2.37 ± 0.17	2.62 ± 0.45	2.60 ± 0.24	2.57 ± 0.21	0.53	0.60	0.41
18:1n	44.59 ± 0.32	42.70 ± 2.04	43.53 ± 0.93	42.68 ± 0.91	0.46	0.09	0.48
20:1n	0.92 ± 0.06	0.93 ± 0.21	0.91 ± 0.12	0.87 ± 0.08	0.68	0.79	0.76
18:1/16:1	18.90 ± 1.52	16.70 ± 3.55	16.82 ± 1.72	16.70 ± 1.31	0.87	0.65	0.67

Values in the graphs are presented as mean and standard deviation (*n* = 3) (one pool of 5 livers per tank * 3). * S = selection, D = diet, S × D = interaction of selection and diet, *p*-values under 0.05 are bolded, ^1^ ∑ Saturated fatty acids include 14:0, 15:0, 16:0, 17:0, 18:0, and 20:0, ^2^ ∑ 16:1n series fatty acids include: 16:1*n-5*, 16:1*n-7*, ^3^ ∑ 18:1n series fatty acids include: 18:1*n-9*, 18:1*n-7*, 18:1*n-5*, ^4^ ∑ 20:1n series fatty acids include: 20:1*n-9*, 20:1*n-7*, 20:1*n-5*.

**Table 6:** Formulation and biochemical composition of the nutritional programming diets fed 1 month prior to egg collection and larval rearing

	100% FO	30% FO–70% VO
Raw Material (%)	(F)	(V)
Meals from marine sources ^1^	50.0	50.0
Sunflower cake	13.2	13.2
Soya cake ^2^	10.0	10.0
Fish oil ^3^	8.0	2.4
Linseed oil ^4^	-	5.6
Wheat	9.9	9.9
Corn gluten 60	7.0	7.0
Drying/wetting	0.9	0.9
Vitamin & mineral premix ^5^	1.0	1.0
Vitamin E powder (50%)	0.1	0.1
**Biochemical Composition**		
**(% of Dry Matter)**		
Moisture	9.1	8.8
Protein (crude)	56.3	56.1
Lipids (crude)	17.2	17.1
Ash	8.6	8.5
**Energy–Gross (MJ kg^−1^)**	21.2	21.2
**Fatty Acids (% of Total Fatty Acids)**		
18:2*n-6*	5.6	9.9
18:3*n-3*	0.9	16.3
20:4*n-6*	0.4	0.3
20:5*n-3*	6.3	4.8
22:6*n-6*	7.1	6.0

^1^ Contains Fish meal NA LT 70, Fish meal SA 68, Feed Service Bremen, Germany.^2^ 48 Hi Pro Solvent Extra. Svane Shipping, Denmark. ^3^ South American fish oil, LDN Fish Oil, Denmark. ^4^ Ch. Daudruy, France. ^5^ Supplied the following vitamins (mg/kg): A 3.8, D 0.05, E 102.4, K3 9.8, B1 2.7, B2 8.3, B6 4.8, B12 0.25, B3 24.8, B5 17.2, folic acid 2.8, H 0.14, C 80; minerals (mg/kg): cobalt 0.94, iodine 0.7, selenium 0.2, iron 32.6. manganese 12, copper 3.2, zinc 67; other (g/kg): taurine 2.45, methionine 0.5, histidine 1.36, cholesterol 1.13. DSM, (Netherlands), Evonik Industries (Germany), Deutsche Lanolin Gesellschaft (Germany).

## Supplementary Materials

Supplementary materials can be found at https://www.mdpi.com/1422-0067/20/24/6250/s1. References [52,53,54,55] are cited in the supplementary materials.
