# Supplementary material for: Effects of Dietary Lipid Composition and Fatty Acid Desaturase 2 Expression in Broodstock Gilthead Sea Bream on Lipid Metabolism-Related Genes and Methylation of the fads2 Gene Promoter in Their Offspring
Source: Int J Mol Sci. 2019 Dec 11;20(24):6250. doi: 10.3390/ijms20246250 (PMC6940931; doi:10.3390/ijms20246250)
Supplement: Supplementary file 1 [file ijms-20-06250-s001.pdf]

Supplementary Material

Table S1. Primers, Genbank accession numbers and reference articles for sequences of target and housekeeping genes**.**

**Gene ***	**Primer Sequence**<br>5′-3′ (F) and 5′-3′ (R)	**GenBank Access No.**	**Reference**
lpl	CGT TGC CAA GTT TGT GAC CTG<br>AGG GTG TTC TGG TTG TCT GC	AY495672	[52]
ppara	TCT CTT CAG CCC ACC ATC CC<br>ATC CCA GCG TGT CGT CTC C	AY590299	[52]
elovl6	GTG CTG CTC TAC TCC TGG TA<br>ACG GCA TGG ACC AAG TAG T	JX975702	[52]
fads2	CGA GAG CCA CAG CAG CAG GGA<br>CGG CCT GCG CCT GAG CAG TT	GQ162822	**
cox2	GAG TAC TGG AAG CCG AGC AC<br>GAT ATC ACT GCC GCC TGA GT	AM296029	[53]
cpt1b	CCA CCA GCC AGA CTC CAC AG<br>CAC CAC CAG CAC CCA CAT ATT TAG	DQ866821	[54]
actb	TCT GTC TGG ATC GGA GGC TC<br>AAG CAT TTG CGG TGG ACG	KY388508	[55]

* Complete gene names; *lpl*: lipoprotein lipase, *ppara*: peroxisome proliferator-activated receptor alpha, *elovl6*: elongation of very long chain fatty acids protein 6, *fads2*: fatty acyl desaturase 2, *cox2*: cyclooxygenase-2, *cpt1*: carnitina palmitoiltransferase I, *ß-act*: beta-actin. ** Submitted without separate published article.
